# Special Issue “*H. pylori* Virulence Factors in the Induction of Gastric Cancer”

**DOI:** 10.3390/toxins10050176

**Published:** 2018-04-26

**Authors:** Jean E. Crabtree, Silja Wessler

**Affiliations:** 1 Leeds Institute Biomedical and Clinical Sciences, St. James’s University Hospital, University of Leeds, Leeds LS9 7TF, UK; 2 Division of Microbiology, Department of Biosciences, Paris-Lodron University of Salzburg, A-5020 Salzburg, Austria

Twenty-five years ago, *Helicobacter pylori* was identified as the causative agent of gastric disorders, ranging from acute inflammation, ulceration of the stomach and duodenum, lymphoma of the MALT (mucosa-associated lymphoid tissue) system and gastric cancer. First assigned as *Campylobacter pylori*, the bacterium was finally renamed *Helicobacter pylori* and defined as the type species of a novel genus after 16S rRNA sequencing. The identification of *H. pylori* revolutionized the treatment of gastric disorders since bacterial eradication can eliminate the root of diseases. The initial contact with *H. pylori* normally occurs in early childhood and infection persists life-long as the immune system cannot efficiently combat the bacterium. As a consequence of the close association of *H. pylori* infection and gastric cancer, the International Agency for Research on Cancer (IARC) classified *H. pylori* as a class-I carcinogen in 1994. Treatment with antibiotics is still the only option to prevent *H. pylori*-associated diseases. Antibiotic therapy has been established as the standard of care in MALT lymphoma patients and can result in complete disappearance of early-stage MALT lymphoma of the stomach. The outcome of the disease is strongly dependent on genetic predispositions of the host, but also on the expression of different bacterial virulence factors, including adhesins, outer membrane proteins (OMPs), proteases, type-IV secretion system (T4SS), cytotoxin-associated gene A (CagA), or vacuolating toxin A (VacA). In this Special Issue, a collection of review and research articles is presented to provide a deep insight into how *H. pylori* interferes with host cells to drive the cells toward inflammatory and carcinogenic responses.

The recent knowledge about *H. pylori*-mediated gastric MALT lymphoma (GML) has been summarized by Floch and colleagues [1]. So far, GML has not been associated with individual *H. pylori* virulence factors. However, a unique Lewis antigen profile in GML strains is possibly implicated in the immune evasion and activation of infiltrating lymphocytes. Similar to MALT lymphoma, the induction and progression of gastric cancer is a multi-step process controlled by a complex interaction between bacterial and host factors. Disruption and depolarization of the gastric epithelium is a hallmark of gastric cancer. Within the complex processes leading to tissue destruction, a controlled proteolysis of epithelial cell surface structure is required which can be directly influenced by *H. pylori*. The proteolytic modulation of extracellular matrix (ECM) proteins, cell surface receptors, membrane-bound cytokines, membrane-bound epidermal growth factor receptor ligands and lateral adhesion molecules involves *H. pylori*-regulated proteases of the host and of bacterial origin. In the review of Posselt et al., the mechanisms of host matrix-metalloproteinases (MMPs), a disintegrin and metalloproteinases (ADAMs), tissue inhibitors of metalloproteinases (TIMPs), and the bacterial secreted proteases Hp0169 and high temperature requirement A (HtrA) were described and how they facilitate the disruption of the epithelial barrier [2]. Among *H. pylori* virulence factors, VacA expression has been connected with *H. pylori* pathogenesis. McClain and colleagues give an overview on epidemiologic studies showing an association between specific *vacA* allelic types and gastric cancer and discuss the mechanisms by which VacA-induced cellular alterations may contribute to the pathogenesis of gastric cancer [3]. A specialized T4SS is exposed by *H. pylori* which translocates CagA into host cells. Independently of CagA injection into epithelial cells, the T4SS is crucially important to induce nuclear factor kappa B (NF-κB) activity; a main driver of cytokine, growth factor, anti-apoptosis regulators or metalloprotease expression. Sokolova and Naumann provide a comprehensive overview about mechanisms and consequences of NF-κB activation in gastric carcinogenesis [4]. OMPs were described as essential molecules to promote CagA translocation into gastric cells via the T4SS of *H. pylori*. The review by Matsuo et al. summarizes the cellular processes through which *H. pylori* utilizes OMPs to colonize the human stomach and how OMPs cooperate with the T4SS [5].

The T4SS-mediated CagA delivery into host cells is considered as a hallmark in *H. pylori* pathogenesis and gastric carcinogenesis. An overview on the epidemiology of CagA-positive *H. pylori* and gastric cancer has been provided by Park et al. [6]. Meta-analyses indicated that CagA positive infections increase the risk of gastric cancer two-fold underlining the importance on population-based *H. pylori* screening and treatment strategies. The molecular mechanism of CagA translocation and the subsequent signal transduction pathways have been reviewed by Tegtmeyer et al. [7]. The T4SS represents an extracellular pilus-like structure exposing CagI, CagL, CagY, and CagA to target the integrin-β1 receptor as a prerequisite for translocation of CagA. Translocated CagA is finally tyrosine phosphorylated by oncogenic host cellular Src and Abl kinase activities. CagA then interacts with several host signalling proteins in phosphorylation-dependent and phosphorylation-independent fashions. The contribution of these interactions to *H. pylori* colonization, signal transduction, and gastric pathogenesis has been included in this review [7]. The interaction of CagA with eukaryotic signalling proteins of the host has been described in more detail by Nishikawa and Hatakeyama. The intrinsically disordered C-terminal part of CagA contains the Glu-Pro-Ile-Tyr-Ala (EPIYA) motif and the CagA multimerization (CM) motif. Dependent on tyrosine phosphorylation, the EPIYA motif binds and deregulates Src homology 2 domain-containing protein tyrosine phosphatase 2 (SHP2), which is important in inducing pro-oncogenic mitogenic signalling and abnormal cell morphology. Through the CM motif, CagA inhibits the kinase activity of polarity regulator partitioning-defective 1b (PAR1b), causing junctional and polarity defects while inducing actin cytoskeletal rearrangements [8]. Further, the research article by Tohidpour and colleagues identified a middle region of CagA which is sufficient to induce its own uptake and rearrangement of the actin cytoskeleton [9].

*H. pylori* is a unique pathogen that colonizes the human stomach persistently. A multitude of virulence factors has been identified in the last decades, which are implicated in pathogenesis. In this special issue, the current knowledge of how these virulence factors interfere with the gastric epithelium providing a comprehensive overview on the molecular and cellular mechanisms leading to chronic inflammation and neoplasia.
